# *Cmfhp* Gene Mediates Fruiting Body Development and Carotenoid Production in *Cordyceps militaris*

**DOI:** 10.3390/biom10030410

**Published:** 2020-03-06

**Authors:** Hai-Wei Lou, Yu Zhao, Bai-Xiong Chen, Ying-Hao Yu, Hong-Biao Tang, Zhi-Wei Ye, Jun-Fang Lin, Li-Qiong Guo

**Affiliations:** 1 Department of Bioengineering, College of Food Science, South China Agricultural University, Guangzhou 510640, China; superharry@163.com (H.-W.L.); bxiong@foxmail.com (B.-X.C.); yuyinghao6@163.com (Y.-H.Y.); zhwye@scau.edu.cn (Z.-W.Y.); 2 College of Food Science and Technology, Henan University of Technology, Zhengzhou 450001, China; zyzhaoyu@yeah.net; 3 Research Center for Micro-Ecological Agent Engineering and Technology of Guangdong Province, Guangzhou 510640, China

**Keywords:** *Cordyceps militaris*, fruiting body, carotenoid, flavohemoprotein, nitric oxide, gene function

## Abstract

*Cordyceps militaris* fruiting bodies contain a variety of bioactive components that are beneficial to the human body. However, the low yield of fruiting bodies and the low carotenoid content in *C. militaris* have seriously hindered the development of the *C. militaris* industry. To elucidate the developmental mechanism of the fruiting bodies of *C. militaris* and the biosynthesis mechanism of carotenoids, the function of the flavohemoprotein-like *Cmfhp* gene of *C. militaris* was identified for the first time. The *Cmfhp* gene was knocked out by the split-marker method, and the targeted gene deletion mutant Δ*Cmfhp* was obtained. An increased nitric oxide (NO) content, no fruiting body production, decreased carotenoid content, and reduced conidial production were found in the mutant Δ*Cmfhp*. These characteristics were restored when the *Cmfhp* gene expression cassette was complemented into the Δ*Cmfhp* strain by the *Agrobacterium tumefaciens*-mediated transformation method. Nonetheless, the *Cmfhp* gene had no significant effect on the mycelial growth rate of *C. militaris*. These results indicated that the *Cmfhp* gene regulated the biosynthesis of NO and carotenoids, the development of fruiting bodies, and the formation of conidia. These findings potentially pave the way to reveal the developmental mechanism of fruiting bodies and the biosynthesis mechanism of carotenoids in *C. militaris*.

## 1. Introduction

*Cordyceps militaris* is a well-known medicinal and edible filamentous fungus. It has been widely used in many countries because it contains many kinds of bioactive components (such as cordycepin, cordycepic acid, polysaccharides, carotenoids, ergosterol, etc.) [1,2,3,4]. These bioactive components endow *C. militaris* with anticancer, antitumor, anti-inflammatory, and antioxidant activities [5,6,7]. In recent years, novel carotenoids with good water solubility and pentostatin with anticancer activity have been found in *C. militaris*, and these findings have increased the market demand for *C. militaris* every year [8,9].

Natural carotenoids play an important role in human health and food coloring. It was suggested that the carotenoid content should be considered as the quality standard of the commercial products of *C. militaris* [10]. *C. militaris*, a potential natural carotenoid resource, cannot meet the market demand because of its low carotenoid content [11,12]. A good way to improve the carotenoid content of *C. militaris* is by genetic engineering. However, there are few reports on carotenoid biosynthesis genes [13]. Therefore, it is necessary to study the genes involved in carotenoid biosynthesis in *C. militaris*.

The main consumer’s demand for *C. militaris* is its fruiting bodies. Hence, scientists have been working to increase the yield of *C. militaris* fruiting bodies [14,15]. However, the developmental mechanism of *C. militaris* fruiting bodies remains unclear. In addition, when *C. militaris* is preserved for a long time or subcultured for many times, its ability to produce fruiting bodies will weaken, or even lose the ability to produce fruiting bodies, which is the degeneration of *C. militaris* [16,17]. The degeneration of *C. militaris* often results in a decrease in the yield of fruiting bodies or in the absence of fruiting bodies [16,17]. Therefore, studying the genes involved in the formation of *C. militaris* fruiting bodies will be helpful to improve the yield of fruiting bodies and will reveal the molecular mechanism of *C. militaris* degeneration.

Light is an essential factor for pigment formation and fruiting body development in *C. militaris* [18]. In our previous experiments, we compared and analyzed the differences between the transcriptome of *C. militaris* mycelia CM10_D cultured under dark conditions and the transcriptome of *C. militaris* mycelia CM10_L cultured under light conditions [19]. We found that the expression level of the *Cmfhp* gene (Gene ID: 18167139) in mycelia CM10_L was significantly higher than that in mycelia CM10_D. The annotation results of the KEGG Orthology (KO) database indicated that the expression product of the *Cmfhp* gene was nitric oxide (NO) dioxygenase. The annotation results of the NCBI non-redundant (NR) protein database showed that the expression product of the *Cmfhp* gene was flavohemoprotein (NCBI accession number: XP_006670328.1). Both annotations indicated that the function of the *Cmfhp* gene was to catalyze the oxidation of NO to nitrate. NO is a signal transduction molecule that plays a variety of important roles in fungi [20]. Previous studies demonstrated that NO regulated the formation of fungal conidia, the growth of mycelia, and the formation of fruiting bodies [21]. In addition, NO is a toxic molecule, and the flavohemoprotein can protect cells from NO toxicity [22]. Therefore, the aim of this study was to study the effects of the *Cmfhp* gene on the NO and carotenoid contents, fruiting body development, and conidial formation of *C. militaris* by gene knockout and gene complementation. This study lays a substantial foundation for revealing the developmental mechanism of fruiting bodies and the biosynthetic pathway of *C. militaris* carotenoids.

## 2. Materials and Methods

### 2.1. Strains and Plasmids

The *C. militaris* strain CM10 (GIM5.271) was maintained on potato dextrose agar (PDA) at 4 °C as a stock. *Escherichia coli* DH5α carried a plasmid pCAMBIA0390-Bar-KOfhp (Figure S1). *Agrobacterium tumefaciens* AGL-1 carried a plasmid pCAMBIA0390-Ben-Comfhp containing the *Cmfhp* gene (Figure S2).

### 2.2. Disruption of the Cmfhp Gene in C. militaris

The *Cmfhp* gene was knocked out by the split-marker method as previously described [23]. The split-marker deletion cassettes were prepared by PCR using the plasmid pCAMBIA0390-Bar-KOfhp as a DNA template. The 5′ split-marker fragment (2292 bp) was amplified with the primers KOfhpU-F and KOfhpU-R. The 3′ split-marker fragment (2801 bp) was amplified with the primers KOfhpD-F and KOfhpD-R (Figure S3 and Table S1). In addition, mononuclear protoplasts of *C. militaris* were prepared by the previously described method [24]. Finally, the split-marker fragments were transformed into mononuclear protoplasts by the previously described transformation method [23]. Colonies that could grow on resistant PDA containing glufosinate ammonium (300 μg/mL) were considered to be putative transformants. The *Cmfhp* gene deletion mutant (Δ*Cmfhp*) was verified by PCR and Southern blot hybridization according to previously reported methods [23]. PCR products were sequenced at Majorbio BioTech Co. (Guangzhou, China) to verify the sequences.

The deletion of the *Cmfhp* gene was further confirmed by quantitative real-time PCR (qRT-PCR) using the *tef1* gene (GenBank: DQ070019) as the internal control gene [25]. Primers *tef1*-F and *tef1*-R (for detecting the *tef1* gene) and primers Qfhp-F and Qfhp-R (for detecting the *Cmfhp* gene) are listed in Table S1. All qRT-PCR was carried out according to previously described methods [26]. The relative expression level of the *Cmfhp* gene was calculated relative to *tef1* expression using the 2^−∆∆CT^ method [27].

### 2.3. Complementation of the Cmfhp Disruption Mutant

The *A. tumefaciens* AGL1-pCAMBIA0390-Ben-Comfhp and conidia of the mutant Δ*Cmfhp* were co-cultured to achieve complementation of the *Cmfhp* gene by the *A. tumefaciens*-mediated transformation (ATMT) method [28]. Colonies that could grow on resistant PDA containing 3 μg/mL of benomyl were considered to be putative transformants. The successful complementary transformants (Δ*Cmfhp-c*) were verified by PCR and qRT-PCR.

### 2.4. Determination of the NO Content

The NO content in *C. militaris* was determined using an NO assay kit (Nanjing Jiancheng Bioengineering Institute, Nanjing, China) according to the manufacturer’s instructions. One gram of fresh *C. militaris* mycelia was ground in 5 mL of 40 mM 4-(2-hydroxyethyl)-1-piperazineethanesulfonic acid (pH 7.2) to assess the NO content. The homogenate was centrifuged at 14,000× *g* for 10 min. The supernatant was used to measure NO [29]. Three biological replicate experiments were performed on each strain of *C. militaris*.

### 2.5. Cultivation of C. militaris Fruiting Bodies

The fruiting bodies of all *C. militaris* strains (CM10, Δ*Cmfhp*, and Δ*Cmfhp-c*) were cultured on rice medium according to a previously described method [14].

### 2.6. Determination of Carotenoid Content

All *C. militaris* strains (CM10, Δ*Cmfhp*, and Δ*Cmfhp-c*) were cultured on PDA for 3 weeks under dark conditions and then 1 week under light conditions. The *C. militaris* mycelia were collected and vacuum freeze-dried. The dried mycelia were used for the determination of carotenoid content according to previously reported methods [10,12].

### 2.7. Growth Rate and Conidial Production

The strains of *C. militaris* (CM10, Δ*Cmfhp*, and Δ*Cmfhp-c*) were inoculated on PDA at 25 °C for 3 weeks before use. A 5-mm disk was punched with a sterilized cutter from the prepared PDA inoculum and transferred to a fresh PDA plate. PDA plates with *C. militaris* inoculum were cultured in the dark at 25 °C. The growth rate was determined by measuring the colony diameter after 3 weeks of incubation [12]. Then, the colonies on PDA medium that had been cultured for 3 weeks were used to determine the production of conidia. Mycelia were scraped from the PDA plates and resuspended in 10 mL of a Tween 80 solution (20%, *w*/*v*). After filtration, the conidial suspensions were counted using a hemocytometer under a microscope [18,30].

### 2.8. Statistical Analysis

All experiments were carried out in triplicate. Data were analyzed by SPSS 22.0 software (SPSS Inc., Chicago, IL, USA). The values are shown as the mean ± standard error. *p*-values less than 0.05 were considered significant.

## 3. Results

### 3.1. Disruption and Complementation of the Cmfhp Gene

**Figure 1:**
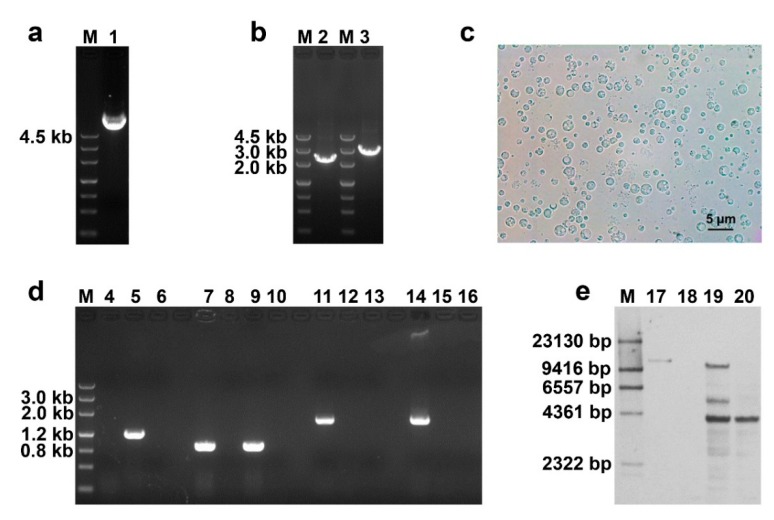
Knockout of the *Cmfhp* gene. (**a**) Extraction of the plasmid pCAMBIA0390-Bar-KOfhp (M: DNA marker; lane 1: plasmid pCAMBIA0390-Bar-KOfhp). (**b**) Preparation of split-marker fragments (M: DNA marker; lane 2: 5′ split-marker fragment; lane 3: 3′ split-marker fragment). (**c**) Preparation of *Cordyceps militaris* protoplasts. (**d**) PCR analysis of the *Cmfhp* gene deletion. Four DNA templates (lanes 4, 7, 11, and 14: genome of the mutant; lanes 5, 8, 12, and 15: genome of wild-type *C. militaris*; lane 9: plasmid pCAMBIA0390-Bar; lanes 6, 10, 13, and 16: ddH_2_O) were used as DNA templates. The 1196 bp fragment of the *Cmfhp* gene was amplified (lanes 4-6). The 890 bp fragment of the *bar* cassette was amplified (lanes 7–10). The upstream flanking sequence (1852 bp) was amplified (lanes 11–13). The downstream flanking sequence (2047 bp) was amplified (lanes 14–16). (**e**) Digested DNA was hybridized with a *bar* probe for Southern blot analysis of PCR-positive mutants (M: DIG-labeled marker; lane 17: *Eco*RV-digested plasmid pCAMBIA0390-Bar-KOFhp, 11,592 bp; lane 18: *Pst*I-digested genome of wild-type *C. militaris*; lanes 19–20: *Pst*I-digested genome of PCR-positive mutants).

Using the plasmid pCAMBIA0390-Bar-KOfhp as a DNA template, the 5′ split-marker fragment and the 3′ split-marker fragment were prepared by PCR amplification (Figure 1a,b). *C. militaris* protoplasts were prepared from mycelia (Figure 1c). Then, 5′ split-marker fragment and the 3′ split-marker fragment were co-transformed into *C. militaris* protoplasts. The results of PCR analysis showed that the *bar* gene was successfully integrated into the *C. militaris* genome and that the *Cmfhp* gene was knocked out (Figure 1d). The results of hybridization with the *bar* probe suggested that there were three hybridization bands in the PCR-positive mutant of lane 19, one hybridization band in the PCR-positive mutant of lane 20, and no hybridization band in wild-type *C. militaris* (Figure 1e).

**Figure 2:**
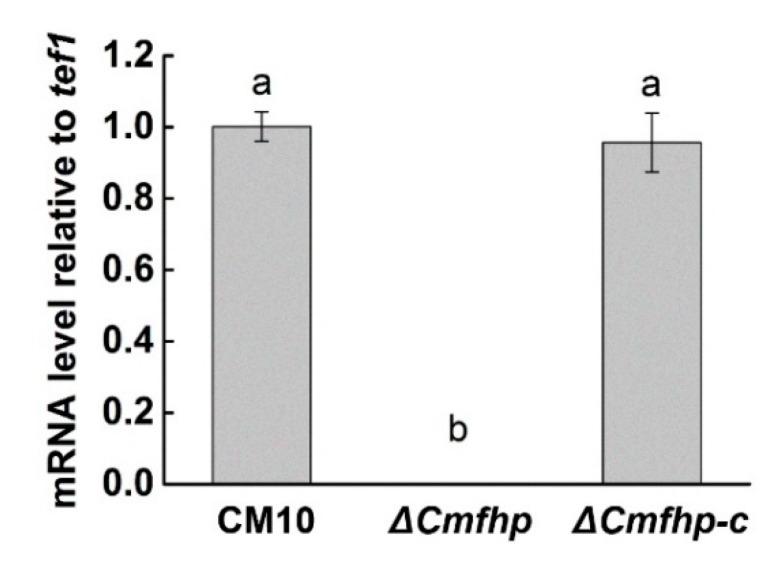
qRT-PCR analysis of the relative expression of the *Cmfhp* gene in *C. militaris* transformants using wild-type *C. militaris* CM10 as a control. Different letters (a,b) indicate significant differences in the relative expression levels of the *Cmfhp* gene.

The mutant corresponding to lane 20 was used for qRT-PCR analysis. The qRT-PCR results demonstrated that the expression of the *Cmfhp* gene was not detected in the mutant Δ*Cmfhp* (Figure 2). Based on these results, the *Cmfhp* gene was successfully knocked out, and the Δ*Cmfhp* strain was used for subsequent experiments.

**Figure 3:**
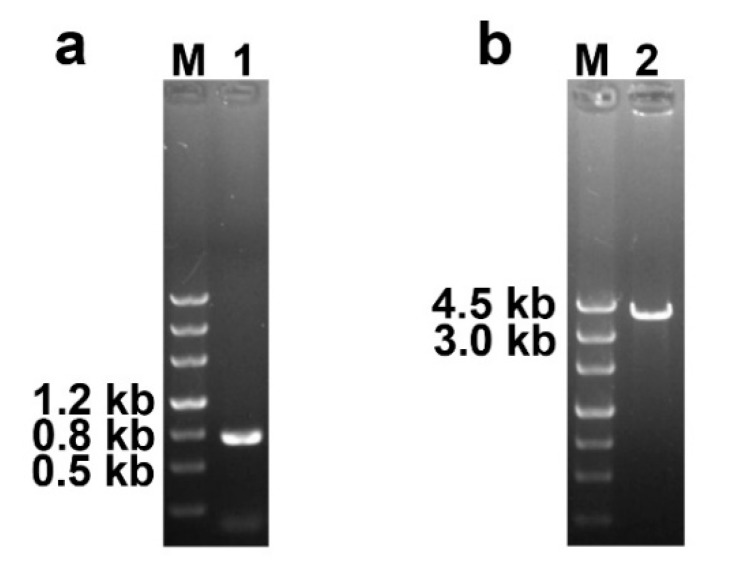
PCR analysis of the complementation of the *Cmfhp* gene. (**a**) PCR amplification of the *ben* gene in complementary transformants (M: DNA marker; lane 1: 754 bp fragment of the *ben* cassette). (**b**) PCR amplification of the *Cmfhp* gene in complementary transformants (M: DNA marker; lane 2: the *Cmfhp* cassette).

The results of PCR amplification of the *ben* gene and the *Cmfhp* gene in the complementary transformants are shown in Figure 3. The sequencing results of the PCR products confirmed that the *ben* gene and the *Cmfhp* gene were successfully integrated into the genome of the Δ*Cmfhp* mutant. The results of qRT-PCR analysis indicated that there was no significant difference in the relative expression level of the *Cmfhp* gene between the wild-type *C. militaris* CM10 and the complementary transformant, which indicated that the *Cmfhp* gene was successfully complemented to the Δ*Cmfhp* mutant and could be expressed in the complementary transformant (Δ*Cmfhp-c*) (Figure 2).

### 3.2. Effect of the Cmfhp Gene on the NO Content in C. militaris

**Figure 4:**
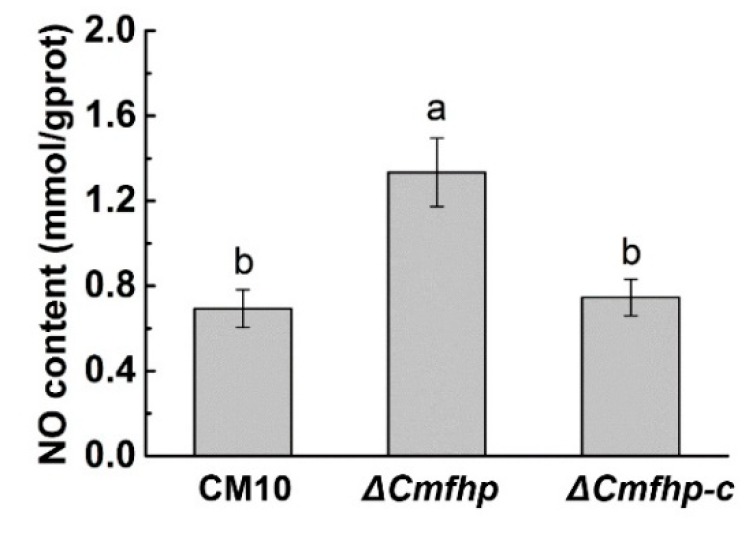
Analysis of the nitric oxide (NO) content in *C. militaris*. Different letters (a,b) indicate significant differences in the NO content.

Compared with wild-type *C. militaris* CM10, *C. militaris* Δ*Cmfhp* contained more NO, which might be due to the deletion of the *Cmfhp* gene. Moreover, the NO content in the complementary strain Δ*Cmfhp-c* was restored to the levels in wild-type *C. militaris* CM10 (Figure 4). Therefore, the expression product of the *Cmfhp* gene could metabolize NO in *C. militaris* and reduce the content of NO in *C. militaris*, and these effects were consistent with the previously reported function of flavohemoprotein [31].

### 3.3. Effect of the Cmfhp Gene on the Development of C. militaris Fruiting Bodies

**Figure 5:**
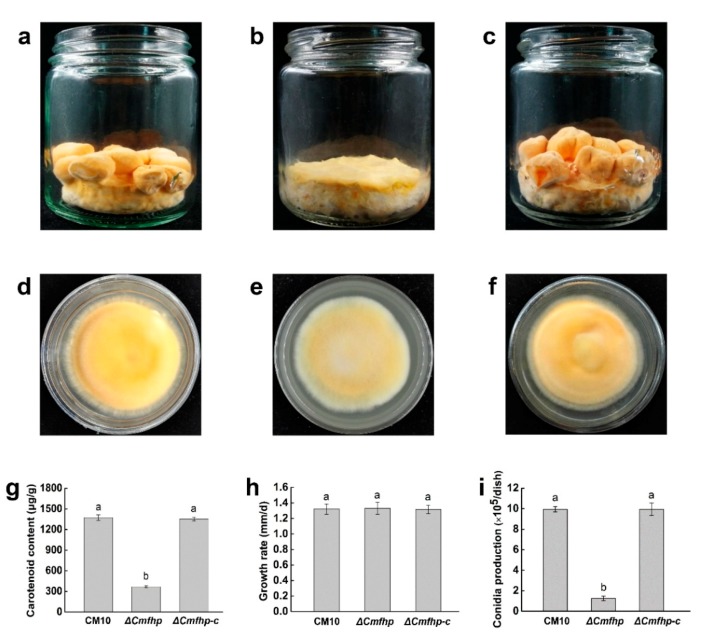
Phenotypic analysis of *C. militaris*. (**a**) *C. militaris* CM10 cultured on rice medium. (**b**) *C. militaris* Δ*Cmfhp* cultured on rice medium. (**c**) *C. militaris* Δ*Cmfhp-c* cultured on rice medium. (**d**) *C. militaris* CM10 cultured on potato dextrose agar (PDA) medium. (**e**) *C. militaris* Δ*Cmfhp* cultured on PDA medium. (**f**) *C. militaris* Δ*Cmfhp-c* cultured on PDA medium. (**g**) Analysis of the carotenoid content in *C. militaris*. (**h**) Analysis of the growth rate of *C. militaris*. (**i**) Analysis of conidial production of *C. militaris*. Different letters (a,b) indicate significant differences.

The fruiting bodies of wild-type *C. militaris* CM10 cultured on rice medium were irregular lumps in shape and were orange in color (Figure 5a). However, *C. militaris* Δ*Cmfhp* cultured on rice medium did not produce fruiting bodies (Figure 5b). When the *Cmfhp* gene was complemented to the mutant Δ*Cmfhp*, the complementary strain Δ*Cmfhp-c* had a restored ability to produce fruiting bodies. Moreover, the fruiting bodies of the complementary strain Δ*Cmfhp-c* were still irregular lumps in shape and were orange in color (Figure 5c). These results suggested that the *Cmfhp* gene was involved in the development of *C. militaris* fruiting bodies.

### 3.4. Effect of the Cmfhp Gene on the Carotenoid Content of C. militaris

The *C. militaris* strains CM10, Δ*Cmfhp*, and Δ*Cmfhp-c* were all cultured on PDA medium (Figure 5d–f). The *C. militaris* strains CM10 and Δ*Cmfhp-c* were all orange in color; however, the color of the *C. militaris* strain Δ*Cmfhp* was light yellow. These results implied that the *Cmfhp* gene regulated the production of *C. militaris* pigments. In addition, the analysis of carotenoid content showed that the carotenoid content of *C. militaris* CM10 was not significantly different from that of *C. militaris* Δ*Cmfhp-c* but was significantly higher than that of *C. militaris* Δ*Cmfhp* (Figure 5g). Therefore, we believe that the *Cmfhp* gene regulated the production of carotenoids in *C. militaris*.

### 3.5. Effect of the Cmfhp Gene on the Growth Rate and Conidial Production of C. militaris

The analysis results of the mycelial growth rate showed that there was no significant difference in the growth rates of the *C. militaris* strains CM10, Δ*Cmfhp*, and Δ*Cmfhp-c* (Figure 5h). This indicated that the *Cmfhp* gene had no significant effect on the growth of *C. militaris*. However, the conidial production of the mutant Δ*Cmfhp* was significantly lower than that of the wild-type *C. militaris*, and there was no significant difference in the conidial production between the wild-type strain CM10 and the complementary strain Δ*Cmfhp-c* (Figure 5i). These results demonstrated that the *Cmfhp* gene significantly affected the conidial production of *C. militaris*.

## 4. Discussion

Light is a necessary condition for the production of *C. militaris* fruiting bodies and pigments [18]. The *Cmfhp* gene of *C. militaris* was significantly upregulated after being treated with light. In the present study, the function of the *Cmfhp* gene in *C. militaris* was studied for the first time by gene knockout and gene complementation. We found that the *Cmfhp* gene not only affected the formation of *C. militaris* fruiting bodies and conidia but also regulated the production of NO and carotenoids. However, the *Cmfhp* gene had no significant effect on the growth rate of *C. militaris* mycelia.

Flavohemoprotein, a NO dioxygenase, is capable of oxidizing NO to nitrate using oxygen [22]. This enzymatic conversion thus protects the cell from toxic NO and from other damaging NO-derived reactive nitrogen species [22]. The deletion of the *Cmfhp* gene led to an accumulation of more NO in the Δ*Cmfhp* mutant, which might be due to the absence of the flavohemoprotein (encoded by the *Cmfhp* gene) that could catalyze the oxidation of NO to nitrate. In addition, NO produced at sufficient levels directly or indirectly damages critical cell processes [32]. Hence, excessive NO might be toxic to the development and metabolism of *C. militaris*.

The consumer’s demand for *C. militaris* is mainly for its fruiting bodies. However, the developmental mechanism of *C. militaris* fruiting bodies is poorly understood. Although *C. militaris* is a heterothallic ascomycetous fungus, it can still produce fruiting bodies without an opposite mating-type partner [33]. Previous studies have also shown that the mating-type is not the decisive factor for the production of *C. militaris* fruiting bodies [34,35]. Therefore, it is necessary to study the key genes involved in the formation of *C. militaris* fruiting bodies. In this study, the wild-type *C. militaris* CM10 with a single mating-type gene (*MAT 1-1*) can stably produce irregular lumpy fruiting bodies. The phenotypic analysis of the fruiting bodies of *C. militaris* strains (CM10, Δ*Cmfhp*, and Δ*Cmfhp-c*) revealed that the *Cmfhp* gene was a key gene affecting the formation of *C. militaris* fruiting bodies. It has been previously reported that NO regulates the formation of fungal fruiting bodies [21]. Therefore, we believe that the loss of the ability to produce fruiting bodies of the Δ*Cmfhp* strain may be due to its high NO content.

Carotenoids are secondary metabolites produced by *C. militaris* cultured under the light. However, the biosynthetic pathway of *C. militaris* carotenoids is still unknown, and there are few reports on the genes involved in the biosynthesis of *C. militaris* carotenoids. A putative carotenoid biosynthetic pathway for *C. militaris* was proposed, but 11 genes involved in this putative pathway were not significantly differentially expressed between the *C. militaris* strain 498 and the *C. militaris* strain 505 after light irradiation [13]. Although three types of geranylgeranyl diphosphate synthase genes in *C. militaris* were cloned, their functions have yet to be identified [36]. It is noteworthy that the other two key enzymes (phytoene synthetase and phytoene dehydrogenase) were not found in the genome of *C. militaris* [13,18]. The induction of carotenoids was completely different between *C. militaris* and *Neurospora crassa* [13,18]. Therefore, it is a great challenge to characterize the carotenoid biosynthetic pathway in *C. militaris*. In this study, the *Cmfhp* gene was identified to have a significant effect on the biosynthesis of carotenoids in *C. militaris*. The carotenoid content of the Δ*Cmfhp* strain was lower than that of wild-type *C. militaris* CM10, while the NO content of the Δ*Cmfhp* strain was significantly higher than that of wild-type *C. militaris* CM10. It has been reported that NO could downregulate the synthesis of carotenoids in *Chlamydomonas reinhardtii* [37]. Therefore, we believed that the excessive NO in the Δ*Cmfhp* strain inhibited the biosynthesis of carotenoids.

NO inhibited the mycelial growth of *Aspergillus niger*, *Monilinia fructicola*, *Penicillium italicum*, and *N. crassa* [21,38]. In the present study, although the NO content in the Δ*Cmfhp* strain was higher than that in wild-type *C. militaris* CM10, there was no significant difference in their growth rate. This might be due to the different tolerance thresholds of different fungi to NO, and the NO content in the Δ*Cmfhp* strain might not reach the level that inhibits the growth of *C. militaris* mycelia. It has also been reported that NO inhibited the conidial production of *N. crassa* [21,39]. In this study, the conidial production in *C. militaris* (CM10, Δ*Cmfhp*, and Δ*Cmfhp-c*) was negatively correlated with the content of NO. This suggests that the conidial production of *C. militaris* may be inhibited by NO.

Flavohemoprotein could detoxify NO in *A. fumigatus* [31]. However, the detoxification mechanism of NO in *C. militaris* is still unknown. Therefore, the following aspects will need to be studied: (1) elucidating the formation mechanism of NO in *C. militaris*; (2) revealing the expression mechanism of the *Cmfhp* gene in *C. militaris*; (3) clarifying the NO detoxification mechanism of the *Cmfhp* gene; (4) elucidating the regulation mechanism of NO on the development of fruiting bodies and the metabolism of bioactive ingredients in *C. militaris*. The identification of the function of the *Cmfhp* gene in this study will also help to reveal the developmental mechanism of fruiting bodies and the biosynthesis mechanism of carotenoids in *C. militaris*.

## 5. Conclusions

The function of the *Cmfhp* gene in *C. militaris* was identified by gene knockout and gene complementation for the first time, and the target gene deletion mutant Δ*Cmfhp* and the target gene complementary strain Δ*Cmfhp-c* were obtained. The deletion of the *Cmfhp* gene resulted in an increase in NO content, the loss of the ability to produce fruiting bodies, a decrease in the carotenoid content, and a reduction in conidial production in the Δ*Cmfhp* mutant. However, the deletion of the *Cmfhp* gene had no significant effect on the mycelial growth rate of *C. militaris*. The identification of the function of the *Cmfhp* gene will be helpful to reveal the developmental mechanism of *C. militaris* fruiting bodies and the metabolic regulation mechanism of carotenoids in *C. militaris*.

## Supplementary Materials

The following are available online at https://www.mdpi.com/2218-273X/10/3/410/s1, Table S1: Oligonucleotide primer sequences used in this study, Figure S1: Construction of the vector pCAMBIA0390-Bar-KOfhp, Figure S2: Construction of the vector pCAMBIA0390-Ben-Comfhp, Figure S3: Schematic diagram for preparing split-marker fragments and deletion of the *Cmfhp* gene.
