# Supplementary material for: Cmfhp Gene Mediates Fruiting Body Development and Carotenoid Production in Cordyceps militaris
Source: Biomolecules. 2020 Mar 6;10(3):410. doi: 10.3390/biom10030410 (PMC7175373; doi:10.3390/biom10030410)
Supplement: Supplementary file 1 [file biomolecules-10-00410-s001.pdf]

biomolecules

MDPI

Supplementary Materials

Cmfhp Gene Mediates Fruiting Body Development and Carotenoid Production in *Cordyceps militaris*

Hai-Wei Lou [1,2], Yu Zhao [2], Bai-Xiong Chen [1], Ying-Hao Yu [1], Hong-Biao Tang [1], Zhi-Wei Ye [1], Jun-Fang Lin [1,3,*] and Li-Qiong Guo [1,3,*]

1 Department of Bioengineering, College of Food Science, South China Agricultural University, Guangzhou 510640, China
2 College of Food Science and Technology, Henan University of Technology, Zhengzhou 450001
3 Research Center for Micro-Ecological Agent Engineering and Technology of Guangdong Province, Guangzhou 510640, China
* Correspondence: linjf@scau.edu.cn (J.-F.L.); guolq@scau.edu.cn (L.-Q.G.)

Table S1. Oligonucleotide primer sequences used in this study.

Primer Name	Primer Sequence (5' to 3')	Amplification purpose
KOfhpU-F	GGATCCGCGCGTGTCAATGCTGAC	Primers for amplifying the 5′ split-marker fragment (2292 bp)
KOfhpU-R	CCGATGACAGCGACCACGCTCTTG	
KOfhpD-F	CCACTACATCGAGACAAGCACGGTCAACTTC	Primers for amplifying the 3′ split-marker fragment (2801 bp)
KOfhpD-R	CGGTGCTTCTCCTATGGGCTTTGATACCATTG	
Bar-F	GCTCGAAGTGTGACTCTTATTAGCAGAC	Primers for amplifying an 890 bp fragment of the *bar* cassette
Bar-R	GCTTGGACAAATGAACGTATCTTATCGAG	
JCfhp-F	CGAGACCATTACGCGCATCTTCTAC	Primers for amplifying an 1196 bp fragment of the *Cmfhp* gene
JCfhp-R	GCTCGAGGTGAATCTTGTCCTCGTT	
JCfhpU-F	GTCGGAGATGCTCTATTCTTATCCACATCT	Primers used to verify the replacement of the upstream end of the *Cmfhp* gene
JCfhpU-R	CGTTCCTGTCTGCTAATAAGAGTCACAC	
JCfhpD-F	CCTACAGGACACACATTCATCGTAGGTAT	Primers used to verify the replacement of the downstream end of the *Cmfhp* gene
JCfhpD-R	GCTGTTAGTTCTGATACCTCTCACAACTCT	
probe-F	TGCACCATCGTCAACCACTACATC	Primers for amplifying a 433 bp fragment of the *bar* gene. The 433 bp fragment was used to prepare the *bar* probe
probe-R	GCTGCCAGAAACCCACGTCAT	
tef1-F	GTCAAGGAAATCCGTCGTGGTAA	Primers for detecting the *tef1* gene expression
tef1-R	GCAGGCGATGTGAGCAGTGTG	
Qfhp-F	CCTTTGCTATCCATGCTCGAC	Primers for detecting the *Cmfhp* gene expression
Qfhp-R	CGACGTTGCTAAGGAAGACC	
CmfhpC-F	GCCTCAACACGACGTACACGGTGTC	Primers for amplifying the entire *Cmfhp* gene
CmfhpC-R	GCAACGAACACACCAAACGGTTCTTCGTAT	
JCBen-F	GGTGCTGCTTTCTGGTACGTCGTCAT	Primers for amplifying a 754 bp fragment of the *ben* gene
JCBen-R	GTCTCGTCGGAGTTCTCAACGAGCTGAT	

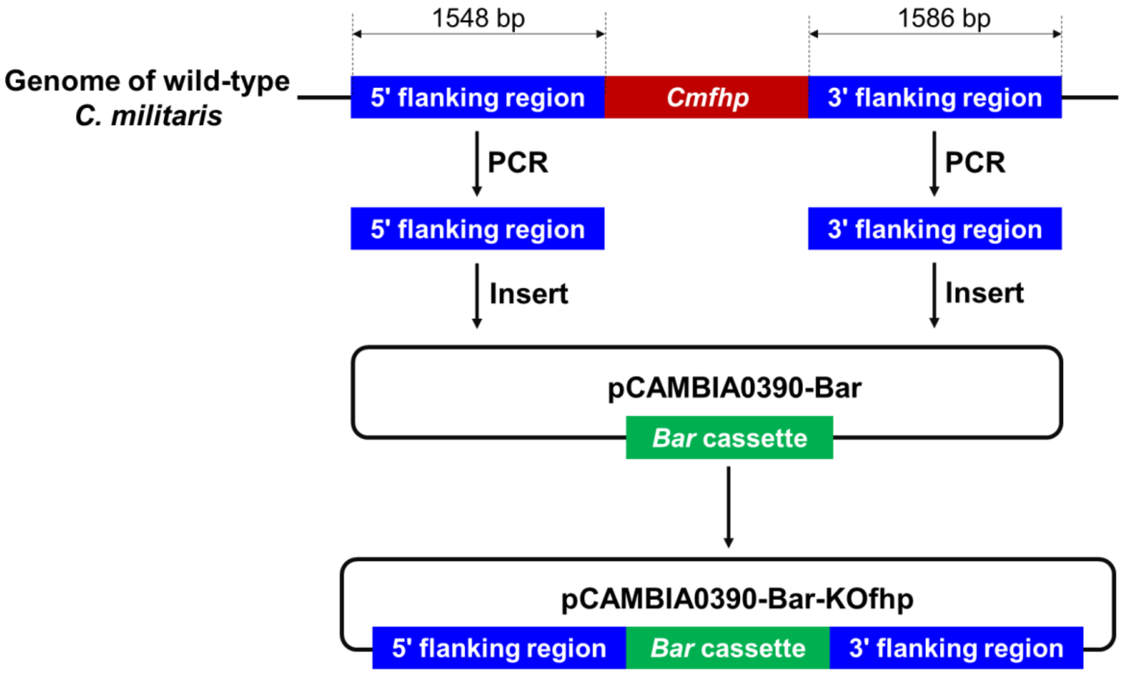

Figure S1. Construction of the vector pCAMBIA0390-Bar-KOfhp. The *bar* cassette contains a phosphinothricin acetyltransferase gene (*bar*) under the control of *Aspergillus nidulans trpC* promoter and *trpC* terminator.

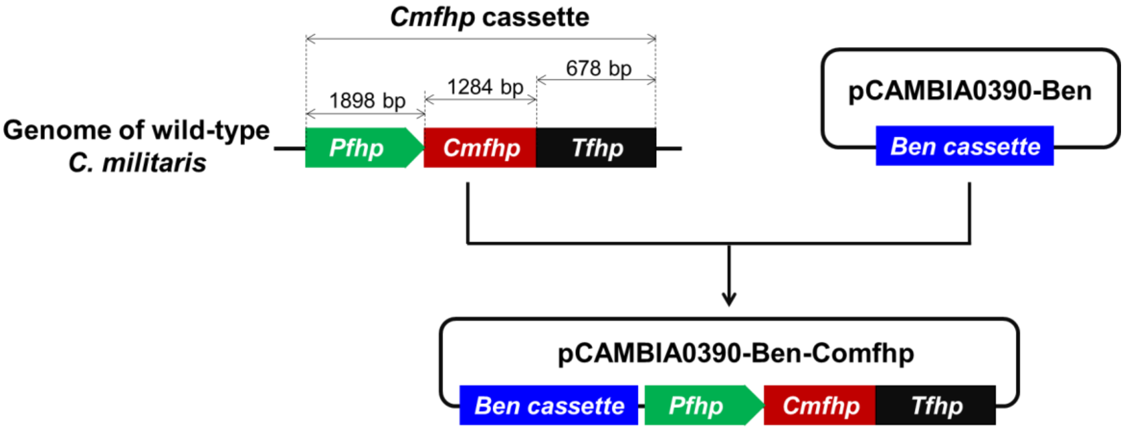

Figure S2. Construction of the vector pCAMBIA0390-Ben-Comfhp. *Pfhp*, promoter of the *Cmfhp* gene; *Tfhp*, terminator of the *Cmfhp* gene. The *ben* cassette contains the benomyl resistant gene (*ben*), the promoter *Pben*, and the terminator *Tben*.

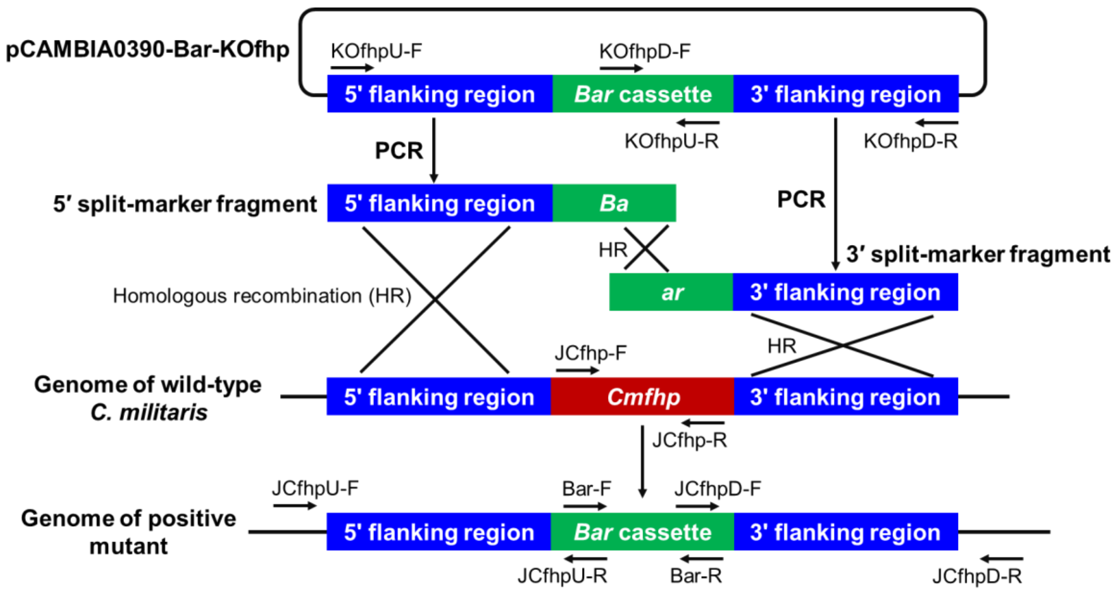

Figure S3. Schematic diagram for preparing split-marker fragments and deletion of the *Cmfhp* gene.
